# Supplementary material for: Performance Evaluation of Multiplex Molecular Syndromic Panel vs. Singleplex PCR for Diagnosis of Acute Central Nervous System Infections
Source: Microorganisms. 2025 Apr 13;13(4):892. doi: 10.3390/microorganisms13040892 (PMC12029859; doi:10.3390/microorganisms13040892)
Supplement: Supplementary file 1 [file microorganisms-13-00892-s001.zip › microorganisms-3545001-supplementary.pdf]

microorganisms

MDPI

Supplementary Material

Table S1. Individual limit of detection values for each QIA/ME viral target.

Target	LoD range	Units
HSV-1	281-338	TCID50/mL
HSV-2	12.6-28.1	TCID50/mL
EV A	3.79-160	TCID50/mL
EV B	43.6-89.1	TCID50/mL
EV C	4.99-15.80	TCID50/mL
EV D	49.9-506	TCID50/mL
HHV-6	31300-72900	cp/mL
VZV	171	cp/mL
VZV	500	TCID 50/mL
HPeV	33.8-1070	TCID 50/mL

LoD - Limit of Detection; TCID50 - Median Tissue Culture Infectious Dose.

Table S2. Bacteria/Yeasts results by routine reference method(s) and QIA/ME.

Sample Code	Positivity by reference method(s) – Culture testing/Antigen testing/ Microscopy	QIA/ME Ct
QIAMEB01	*S. pneumoniae*	18.3
QIAMEB02	*L. monocytogenes*	28.5
QIAMEB03	*S. pneumoniae*	32.1
QIAMEB04 [A]	*C. neoformans*	26.4
QIAMEB05	*N. meningitidis*	13.9
QIAMEB06	*S. pneumoniae*	26.1
QIAMEB07	*S. pneumoniae*	27.3
QIAMEB08	*S. pneumoniae*	17.0
QIAMEB09	*S. agalactiae*	26.2
QIAMEB10	*S. pneumoniae*	21.3
QIAMEB11	*S. pneumoniae*	16.7
QIAMEB12	*N. meningitidis*	22.1
QIAMEB13	*S. pneumoniae*	28.1
QIAMEB14	*S. pyogenes*	27.3
QIAMEB15	*C. neoformans*	21.0
QIAMEB16	*S. pneumoniae*	33.8
QIAMEB17 [B]	*S. agalactiae*	19.8
QIAMEB18	*N. meningitidis*	27.6
QIAMEB19	*L. monocytogenes*	35.7
QIAMEB20 [C]	*H. influenzae*	31.8

[A,B,C] Viral-bacterial/yeast coinfections corresponding viral codes: [A] QIAMEV096 ; [B] QIAMEV020; [C] QIAMEV045.

Table S3. Concordant results between singleplex PCR and QIA/ME (HSV-1).

Sample code	Singleplex PCR		QIA/ME
	Copies/mL	Log_{10} copie/mL	Ct
QIAMEV090	249	2.396199	34.6
QIAMEV076	615	2.788875	35.1
QIAMEV133	651	2.813581	35.3
QIAMEV005	651	2.813581	36.5
QIAMEV070	961	2.982723	35.7
QIAMEV040	968	2.985875	34.0
QIAMEV039	1278	3.106531	34.7
QIAMEV108	1410	3.149219	34.1
QIAMEV149	2190	3.340444	36.8
QIAMEV072	2548	3.406199	32.9
QIAMEV139	5796	3.763128	31.1
QIAMEV013	6343	3.802295	29.9
QIAMEV148	8793	3.944137	34.0
QIAMEV129	9363	3.971415	31.3
QIAMEV036	9650	3.984527	31.7
QIAMEV030	10715	4.029992	31.0
QIAMEV087	10764	4.031974	32.8
QIAMEV042	11456	4.059033	30.8
QIAMEV078	24324	4.386035	31.8
QIAMEV069	25886	4.413065	29.8
QIAMEV132	35949	4.555687	29.3
QIAMEV140	73595	4.866848	28.7
QIAMEV016	78194	4.893173	28.9
QIAMEV101	100862	5.003727	27.6
QIAMEV048	109382	5.038946	28.0
QIAMEV047	153906	5.187255	28.7
QIAMEV134	448558	5.651819	25.1
QIAMEV018	725931	5.860895	24.4
QIAMEV080	1219162	6.086061	23.3

Table S4. Discordant results between singleplex PCR and QIA/ME (HSV-1).

Sample code	Singleplex PCR		QIA/ME
	Copies/mL	Log_{10} copie/mL	Ct
QIAMEV023	249	2.396199	0
QIAMEV028	249	2.396199	0
QIAMEV035	249	2.396199	0
QIAMEV096	249	2.396199	0
QIAMEV051	249	2.396199	0
QIAMEV106	249	2.396199	0
QIAMEV094	1143	3.058046	0

Table S5. Concordant results between singleplex PCR and QIA/ME (HSV-2).

Sample code	Singleplex PCR		QIA/ME
	Copies/mL	Log_{10} copie/mL	Ct
QIAMEV109	132	2.120574	36.1
QIAMEV025	249	2.396199	35.6
QIAMEV050	249	2.396199	36.0
QIAMEV112	249	2.396199	36.7
QIAMEV130	1234	3.091315	35.1
QIAMEV027	2123	3.32695	34.4
QIAMEV002	8084	3.907626	32.8
QIAMEV084	9452	3.975524	30.3
QIAMEV055	15290	4.184408	29.5
QIAMEV079	17625	4.246129	29.9
QIAMEV034	44867	4.651927	27.9
QIAMEV009	45726	4.660163	28.3
QIAMEV147	53160	4.725585	31.9
QIAMEV032	66390	4.822103	27.9
QIAMEV150	2707094	6.432503	21.7

Table S6. Concordant results between singleplex PCR and QIA/ME (VZV).

Sample code	Singleplex PCR		QIA/ME
	Copies/mL	Log_{10} copie/mL	Ct
QIAMEV097	249	2.396199	35.0
QIAMEV019	249	2.396199	38.4
QIAMEV077	249	2.396199	38.9
QIAMEV035	409	2.611723	36.6
QIAMEV074	629	2.798651	37.4
QIAMEV022	684	2.835056	37.7
QIAMEV081	1699	3.230193	33.4
QIAMEV064	2774	3.443106	31.9
QIAMEV117	2834	3.4524	34.6
QIAMEV115	2938	3.468052	32.9
QIAMEV114	3001	3.477266	33.7
QIAMEV071	3667	3.564311	32.9
QIAMEV044	3776	3.577032	32.0
QIAMEV001	4286	3.632052	32.1
QIAMEV121	4492	3.65244	36.0
QIAMEV056	5319	3.72583	32.5
QIAMEV007	9946	3.997648	28.6
QIAMEV033	11343	4.054728	31.7
QIAMEV131	12516	4.097466	31.6
QIAMEV103	13369	4.126099	31.3
QIAMEV046	24759	4.393733	29.1
QIAMEV104	24759	4.458578	30.1
QIAMEV037	33004	4.518567	32.2
QIAMEV100	34860	4.542327	28.5
QIAMEV088	35990	4.556182	29.5
QIAMEV122	38636	4.586992	32.6
QIAMEV011	59148	4.77194	28.0
QIAMEV042	76103	4.881402	26.3
QIAMEV060	107133	5.029923	28.4
QIAMEV058	235937	5.372796	27.8
QIAMEV067	322984	5.509181	25.2
QIAMEV135	358869	5.554936	26.3
QIAMEV017	1959775	6.292206	23.8
QIAMEV062	11381923	7.056216	20.5
QIAMEV099	19708524	7.294654	18.6
QIAMEV107	25000000	7.39794	17.1

Table S7. Discordant results between singleplex PCR and QIA/ME (VZV).

Sample code	Singleplex PCR		QIA/ME
	Copies/mL	Log_{10} copie/mL	Ct
QIAMEV119	249	2.396199	0
QIAMEV038	249	2.396199	0
QIAMEV120	249	2.396199	0
QIAMEV010	411	2.613842	0

Table S8. Concordant results between singleplex PCR and QIA/ME (EV).

Sample code	Singleplex PCR		QIA/ME
	Copies/mL	Log_{10} copie/mL	Ct
QIAMEV029	499	2.698101	34.2
QIAMEV054	499	2.698101	34.8
QIAMEV091	499	2.698101	36.8
QIAMEV086	499	2.698101	37.9
QIAMEV014	803	2.904716	35.4
QIAMEV004	972	2.987666	36.0
QIAMEV125	1355	3.131939	38.7
QIAMEV024	2123	3.32695	39.0
QIAMEV095	2444	3.388101	32.2
QIAMEV053	2445	3.388279	36.4
QIAMEV126	2983	3.474653	35.0
QIAMEV138	3001	3.477266	35.4
QIAMEV144	3068	3.486855	36.3
QIAMEV141	3591	3.555215	36.9
QIAMEV003	3591	3.555215	37.2
QIAMEV089	4134	3.61637	38.3
QIAMEV124	7210	3.857935	35.6
QIAMEV006	7210	3.857935	35.8
QIAMEV136	12275	4.089022	33.0
QIAMEV142	14751	4.168821	33.5
QIAMEV113	19393	4.287645	31.2
QIAMEV083	20344	4.308436	33.5
QIAMEV137	50923	4.706914	34.0
QIAMEV143	74288	4.870919	35.0

Table S9. Discordant results between singleplex PCR and QIA/ME (EV).

Sample code	Singleplex PCR		QIA/ME
	Copies/mL	Log_{10} copie/mL	Ct
QIAMEV085	499	2.698101	0
QIAMEV105	499	2.698101	0
QIAMEV015	1312	3.117934	0
QIAMEV093	4939	3.693639	0

Table S10. Concordant results between singleplex PCR and QIA/ME (HHV-6).

Sample code	Singleplex PCR		QIA/ME
	Copies/mL	Log_{10} copie/mL	Ct
QIAMEV010	249	2.396199	35.5
QIAMEV083	249	2.396199	36.9
QIAMEV059	249	2.396199	38.3
QIAMEV092	319	2.503791	37.4
QIAMEV021	378	2.577492	36.8
QIAMEV110	597	2.775974	35.9
QIAMEV111	634	2.802089	35.2
QIAMEV041	833	2.920645	34.5
QIAMEV123	846	2.92737	37.7
QIAMEV118	889	2.948902	36.1
QIAMEV057	905	2.956649	35.3
QIAMEV082	973	2.988113	35.0
QIAMEV065	1103	3.042576	33.4
QIAMEV045	1312	3.117934	34.8
QIAMEV020	1322	3.121232	35.0
QIAMEV063	1593	3.202216	32.1
QIAMEV049	1820	3.260071	35.4
QIAMEV061	1821	3.26031	35.5
QIAMEV066	1823	3.260787	35.0
QIAMEV007	2184	3.339253	26.0
QIAMEV116	2332	3.367728	32.8
QIAMEV073	3461	3.539201	33.5
QIAMEV102	3845	3.584896	30.4
QIAMEV043	4193	3.622525	33.5
QIAMEV127	6744	3.828918	32.1
QIAMEV012	8222	3.914978	30.8
QIAMEV068	9743	3.988693	32.6
QIAMEV128	20238	4.306168	27.7
QIAMEV098	20238	4.306168	28.8
QIAMEV075	89095	4.949853	28.0

Table S11. Discordant results between singleplex PCR and QIA/ME (HHV-6).

Sample code	Singleplex PCR		QIA/ME
	Copies/mL	Log_{10} copie/mL	Ct
QIAMEV031	249	2.396199	0
QIAMEV084	249	2.396199	0
QIAMEV036	249	2.396199	0
QIAMEV026	249	2.396199	0
QIAMEV052	249	2.396199	0
QIAMEV008	249	2.396199	0
QIAMEV037	249	2.396199	0

Table S12. Concordant results between singleplex PCR and QIA/ME (HPeV).

Sample code	Singleplex PCR	QIA/ME
	Qualitative result	Ct
QIAMEV145	Positive	29.6
QIAMEV146	Positive	33.1
